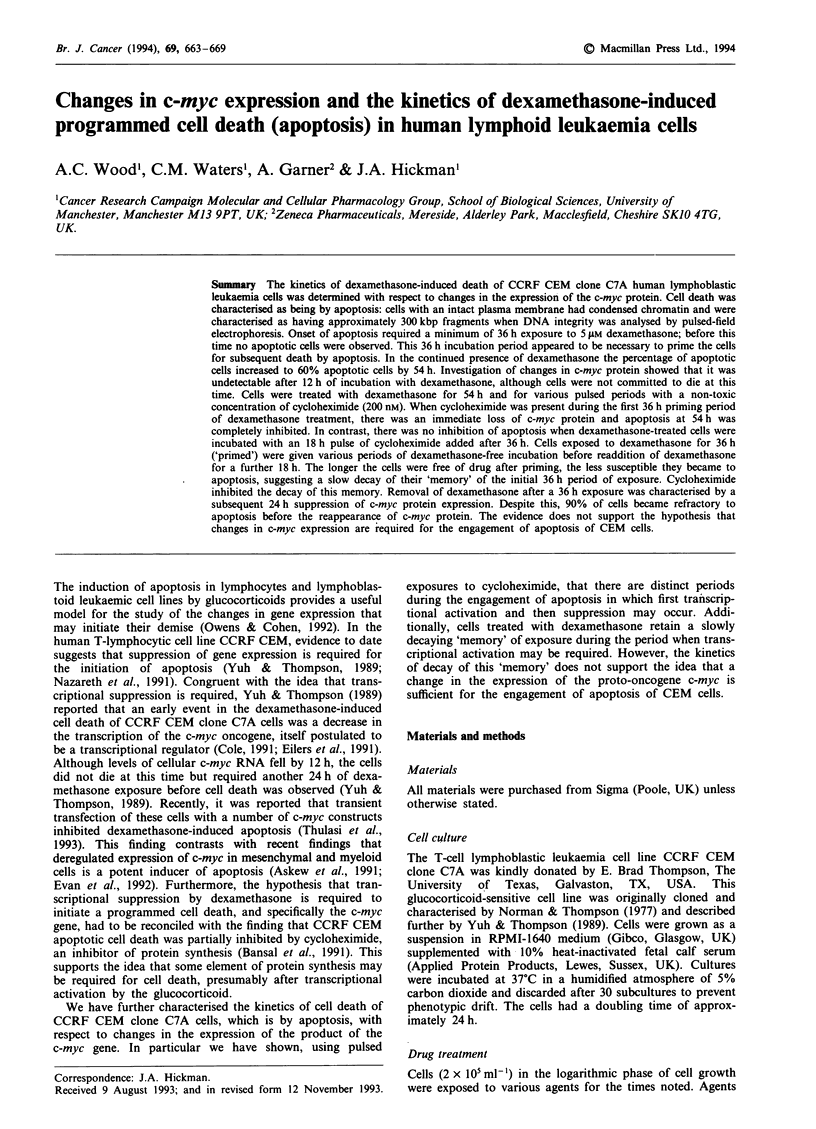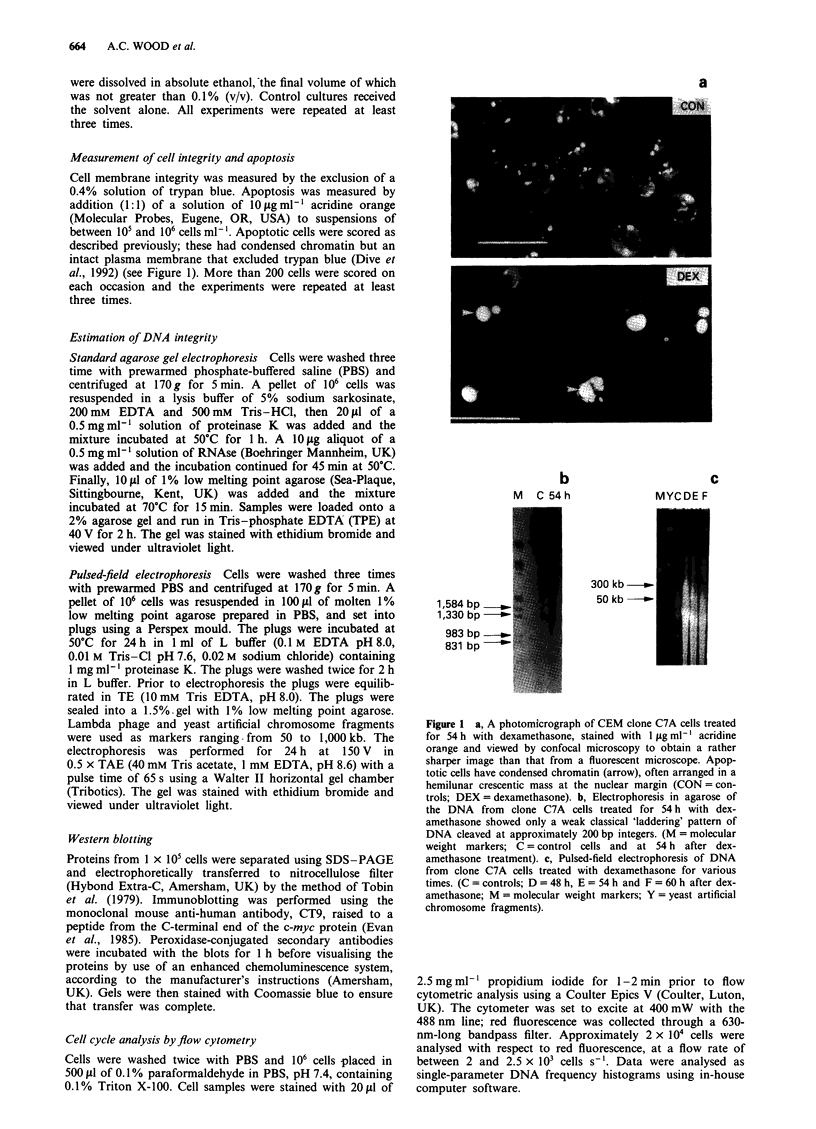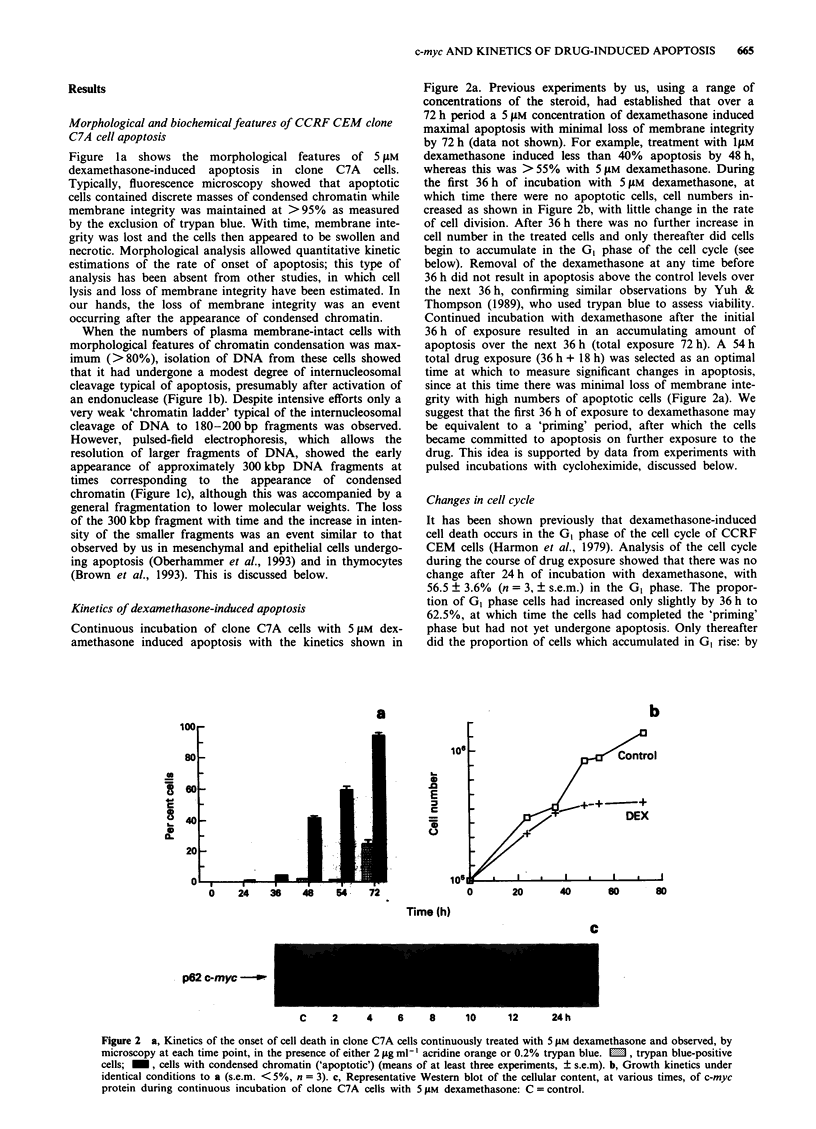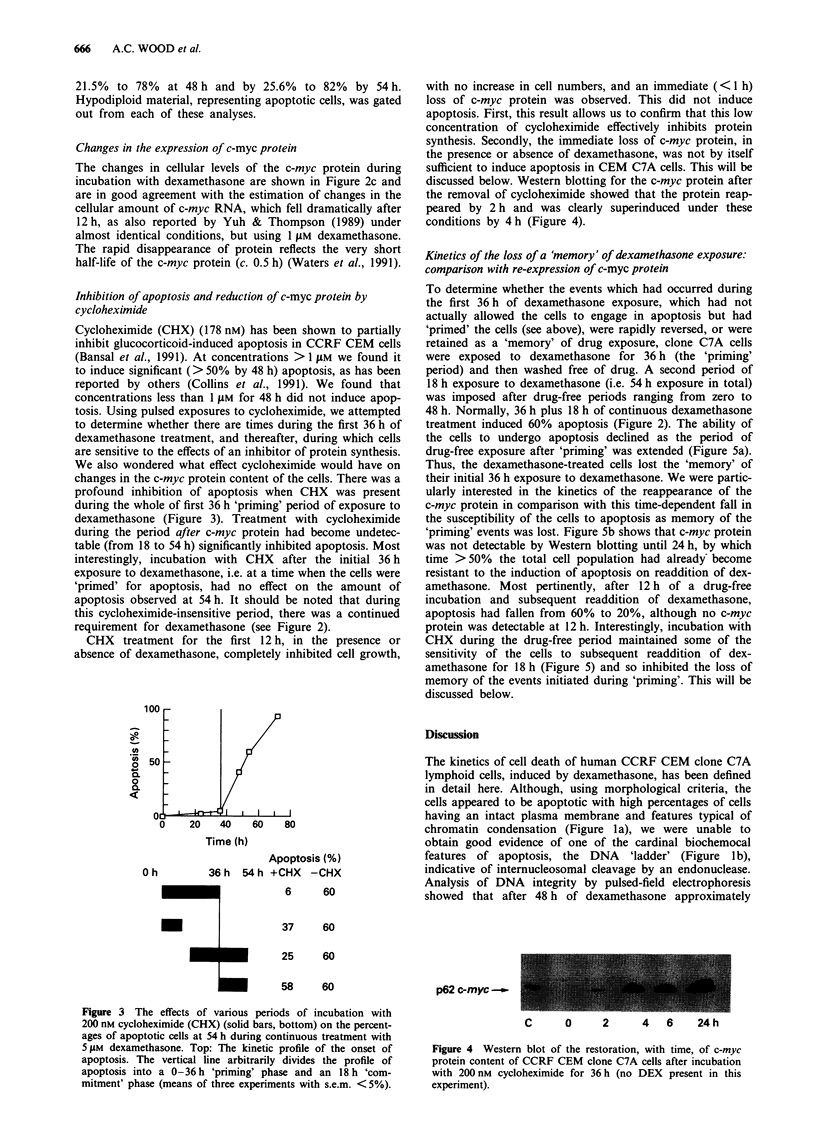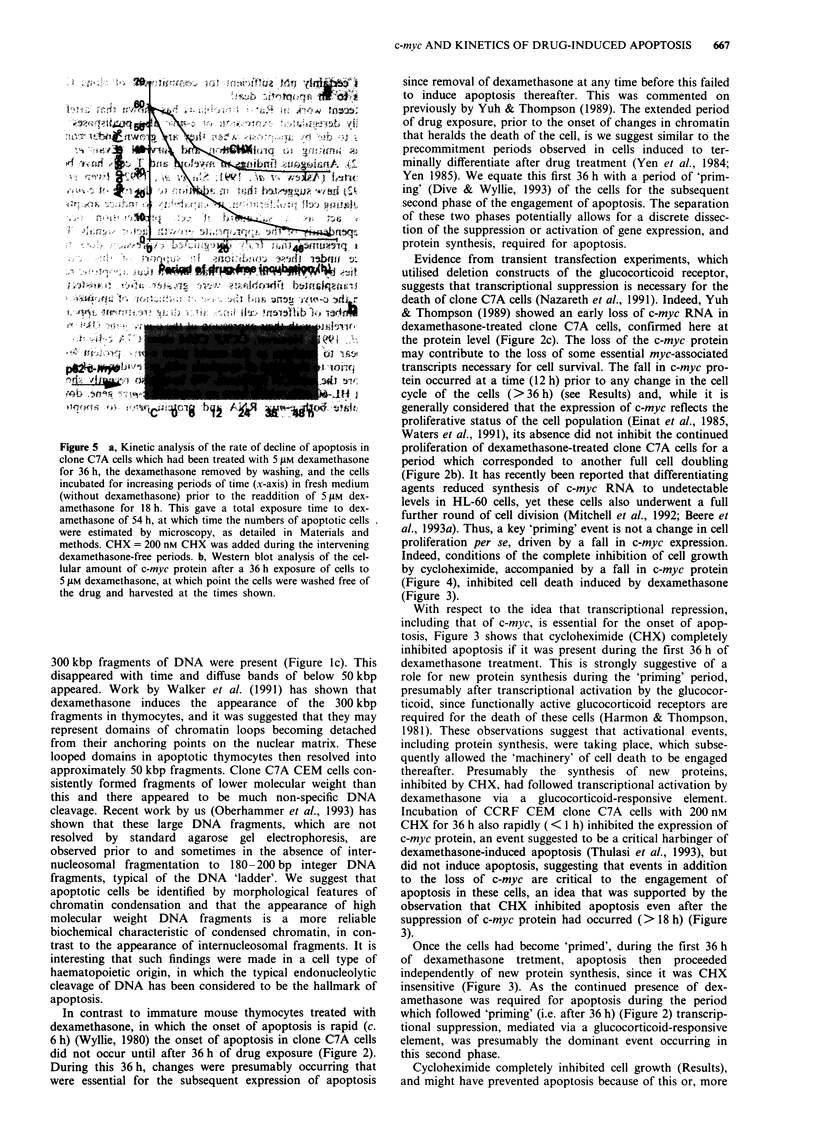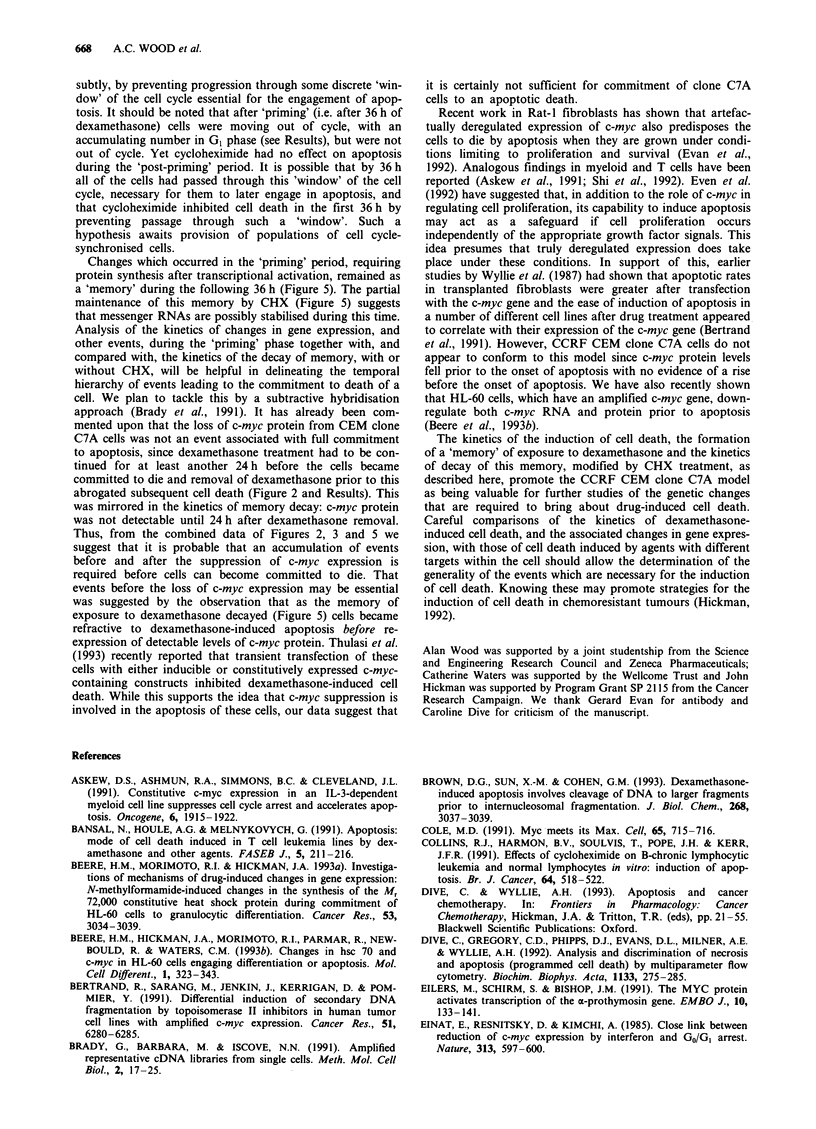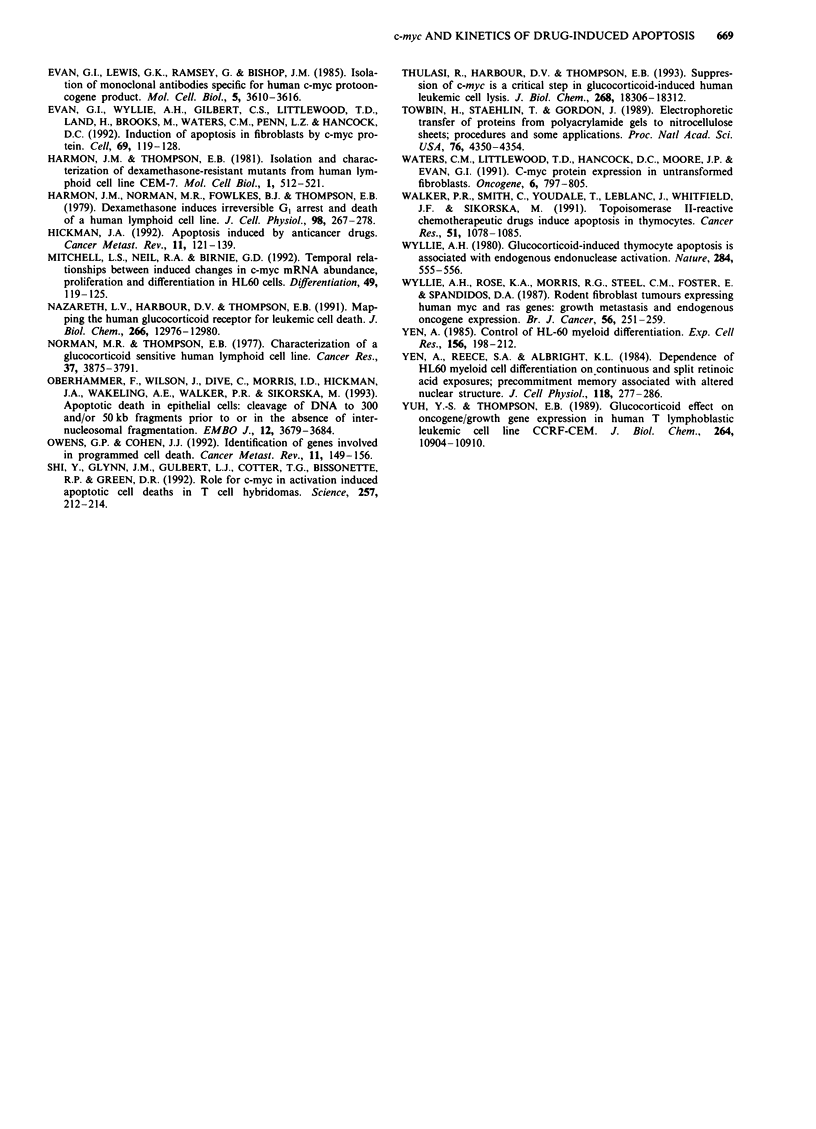# Changes in c-myc expression and the kinetics of dexamethasone-induced programmed cell death (apoptosis) in human lymphoid leukaemia cells.

**DOI:** 10.1038/bjc.1994.128

**Published:** 1994-04

**Authors:** A. C. Wood, C. M. Waters, A. Garner, J. A. Hickman

**Affiliations:** Cancer Research Campaign Molecular and Cellular Pharmacology Group, School of Biological Sciences, University of Manchester, UK.

## Abstract

**Images:**


Br. J. Cancer (1994), 69, 663-669 © Macmillan Press Ltd., 1994

### Changes in c-*myc* expression and the kinetics of dexamethasone-induced programmed cell death (apoptosis) in human lymphoid leukaemia cells

A.C. Wood[1], C.M. Waters[1], A. Garner[2] & J.A. Hickman[1]

[1]*Cancer Research Campaign Molecular and Cellular Pharmacology Group, School of Biological Sciences, University of Manchester, Manchester M13 9PT, UK;* [2]*Zeneca Pharmaceuticals, Mereside, Alderley Park, Macclesfield, Cheshire SK10 4TG, UK.*

**Summary** The kinetics of dexamethasone-induced death of CCRF CEM clone C7A human lymphoblastic leukaemia cells was determined with respect to changes in the expression of the c-*myc* protein. Cell death was characterised as being by apoptosis: cells with an intact plasma membrane had condensed chromatin and were characterised as having approximately 300 kbp fragments when DNA integrity was analysed by pulsed-field electrophoresis. Onset of apoptosis required a minimum of 36 h exposure to 5 μM dexamethasone; before this time no apoptotic cells were observed. This 36 h incubation period appeared to be necessary to prime the cells for subsequent death by apoptosis. In the continued presence of dexamethasone the percentage of apoptotic cells increased to 60% apoptotic cells by 54 h. Investigation of changes in c-*myc* protein showed that it was undetectable after 12 h of incubation with dexamethasone, although cells were not committed to die at this time. Cells were treated with dexamethasone for 54 h and for various pulsed periods with a non-toxic concentration of cycloheximide (200 nM). When cycloheximide was present during the first 36 h priming period of dexamethasone treatment, there was an immediate loss of c-*myc* protein and apoptosis at 54 h was completely inhibited. In contrast, there was no inhibition of apoptosis when dexamethasone-treated cells were incubated with an 18 h pulse of cycloheximide added after 36 h. Cells exposed to dexamethasone for 36 h ('primed') were given various periods of dexamethasone-free incubation before readdition of dexamethasone for a further 18 h. The longer the cells were free of drug after priming, the less susceptible they became to apoptosis, suggesting a slow decay of their 'memory' of the initial 36 h period of exposure. Cycloheximide inhibited the decay of this memory. Removal of dexamethasone after a 36 h exposure was characterised by a subsequent 24 h suppression of c-*myc* protein expression. Despite this, 90% of cells became refractory to apoptosis before the reappearance of c-*myc* protein. The evidence does not support the hypothesis that changes in c-*myc* expression are required for the engagement of apoptosis of CEM cells.

The induction of apoptosis in lymphocytes and lymphoblastoid leukaemic cell lines by glucocorticoids provides a useful model for the study of the changes in gene expression that may initiate their demise (Owens & Cohen, 1992). In the human T-lymphocytic cell line CCRF CEM, evidence to date suggests that suppression of gene expression is required for the initiation of apoptosis (Yuh & Thompson, 1989; Nazareth *et al.*, 1991). Congruent with the idea that transcriptional suppression is required, Yuh & Thompson (1989) reported that an early event in the dexamethasone-induced cell death of CCRF CEM clone C7A cells was a decrease in the transcription of the c-*myc* oncogene, itself postulated to be a transcriptional regulator (Cole, 1991; Eilers *et al.*, 1991). Although levels of cellular c-*myc* RNA fell by 12 h, the cells did not die at this time but required another 24 h of dexamethasone exposure before cell death was observed (Yuh & Thompson, 1989). Recently, it was reported that transient transfection of these cells with a number of c-*myc* constructs inhibited dexamethasone-induced apoptosis (Thulasi *et al.*, 1993). This finding contrasts with recent findings that deregulated expression of c-*myc* in mesenchymal and myeloid cells is a potent inducer of apoptosis (Askew *et al.*, 1991; Evan *et al.*, 1992). Furthermore, the hypothesis that transcriptional suppression by dexamethasone is required to initiate a programmed cell death, and specifically the c-*myc* gene, had to be reconciled with the finding that CCRF CEM apoptotic cell death was partially inhibited by cycloheximide, an inhibitor of protein synthesis (Bansal *et al.*, 1991). This supports the idea that some element of protein synthesis may be required for cell death, presumably after transcriptional activation by the glucocorticoid.

We have further characterised the kinetics of cell death of CCRF CEM clone C7A cells, which is by apoptosis, with respect to changes in the expression of the product of the c-*myc* gene. In particular we have shown, using pulsed exposures to cycloheximide, that there are distinct periods during the engagement of apoptosis in which first transcriptional activation and then suppression may occur. Additionally, cells treated with dexamethasone retain a slowly decaying 'memory' of exposure during the period when transcriptional activation may be required. However, the kinetics of decay of this 'memory' does not support the idea that a change in the expression of the proto-oncogene c-*myc* is sufficient for the engagement of apoptosis of CEM cells.

Correspondence: J.A. Hickman.
Received 9 August 1993; and in revised form 12 November 1993.

### Materials and methods

### Materials

All materials were purchased from Sigma (Poole, UK) unless otherwise stated.

### Cell culture

The T-cell lymphoblastic leukaemia cell line CCRF CEM clone C7A was kindly donated by E. Brad Thompson, The University of Texas, Galvaston, TX, USA. This glucocorticoid-sensitive cell line was originally cloned and characterised by Norman & Thompson (1977) and described further by Yuh & Thompson (1989). Cells were grown as a suspension in RPMI-1640 medium (Gibco, Glasgow, UK) supplemented with 10% heat-inactivated fetal calf serum (Applied Protein Products, Lewes, Sussex, UK). Cultures were incubated at 37°C in a humidified atmosphere of 5% carbon dioxide and discarded after 30 subcultures to prevent phenotypic drift. The cells had a doubling time of approximately 24 h.

### Drug treatment

Cells ($2 \times 10^5$ $ml^{-1}$) in the logarithmic phase of cell growth were exposed to various agents for the times noted. Agents were dissolved in absolute ethanol, the final volume of which was not greater than 0.1% (v/v). Control cultures received the solvent alone. All experiments were repeated at least three times.

*Measurement of cell integrity and apoptosis*

Cell membrane integrity was measured by the exclusion of a 0.4% solution of trypan blue. Apoptosis was measured by addition (1:1) of a solution of $10\,\mu g\,ml^{-1}$ acridine orange (Molecular Probes, Eugene, OR, USA) to suspensions of between $10^5$ and $10^6$ cells $ml^{-1}$. Apoptotic cells were scored as described previously; these had condensed chromatin but an intact plasma membrane that excluded trypan blue (Dive *et al.*, 1992) (see Figure 1). More than 200 cells were scored on each occasion and the experiments were repeated at least three times.

*Estimation of DNA integrity*

*Standard agarose gel electrophoresis* Cells were washed three time with prewarmed phosphate-buffered saline (PBS) and centrifuged at 170 *g* for 5 min. A pellet of $10^6$ cells was resuspended in a lysis buffer of 5% sodium sarkosinate, 200 mM EDTA and 500 mM Tris-HCl, then 20 µl of a $0.5\,mg\,ml^{-1}$ solution of proteinase K was added and the mixture incubated at 50°C for 1 h. A 10 µg aliquot of a $0.5\,mg\,ml^{-1}$ solution of RNAse (Boehringer Mannheim, UK) was added and the incubation continued for 45 min at 50°C. Finally, 10 µl of 1% low melting point agarose (Sea-Plaque, Sittingbourne, Kent, UK) was added and the mixture incubated at 70°C for 15 min. Samples were loaded onto a 2% agarose gel and run in Tris-phosphate EDTA (TPE) at 40 V for 2 h. The gel was stained with ethidium bromide and viewed under ultraviolet light.

*Pulsed-field electrophoresis* Cells were washed three times with prewarmed PBS and centrifuged at 170 *g* for 5 min. A pellet of $10^6$ cells was resuspended in 100 µl of molten 1% low melting point agarose prepared in PBS, and set into plugs using a Perspex mould. The plugs were incubated at 50°C for 24 h in 1 ml of L buffer (0.1 M EDTA pH 8.0, 0.01 M Tris-Cl pH 7.6, 0.02 M sodium chloride) containing $1\,mg\,ml^{-1}$ proteinase K. The plugs were washed twice for 2 h in L buffer. Prior to electrophoresis the plugs were equilibrated in TE (10 mM Tris EDTA, pH 8.0). The plugs were sealed into a 1.5% gel with 1% low melting point agarose. Lambda phage and yeast artificial chromosome fragments were used as markers ranging from 50 to 1,000 kb. The electrophoresis was performed for 24 h at 150 V in 0.5 × TAE (40 mM Tris acetate, 1 mM EDTA, pH 8.6) with a pulse time of 65 s using a Walter II horizontal gel chamber (Tribotics). The gel was stained with ethidium bromide and viewed under ultraviolet light.

*Western blotting*

Proteins from $1 \times 10^5$ cells were separated using SDS-PAGE and electrophoretically transferred to nitrocellulose filter (Hybond Extra-C, Amersham, UK) by the method of Tobin *et al.* (1979). Immunoblotting was performed using the monoclonal mouse anti-human antibody, CT9, raised to a peptide from the C-terminal end of the c-*myc* protein (Evan *et al.*, 1985). Peroxidase-conjugated secondary antibodies were incubated with the blots for 1 h before visualising the proteins by use of an enhanced chemoluminescence system, according to the manufacturer's instructions (Amersham, UK). Gels were then stained with Coomassie blue to ensure that transfer was complete.

*Cell cycle analysis by flow cytometry*

Cells were washed twice with PBS and $10^6$ cells placed in 500 µl of 0.1% paraformaldehyde in PBS, pH 7.4, containing 0.1% Triton X-100. Cell samples were stained with 20 µl of $2.5\,mg\,ml^{-1}$ propidium iodide for 1-2 min prior to flow cytometric analysis using a Coulter Epics V (Coulter, Luton, UK). The cytometer was set to excite at 400 mW with the 488 nm line; red fluorescence was collected through a 630-nm-long bandpass filter. Approximately $2 \times 10^4$ cells were analysed with respect to red fluorescence, at a flow rate of between 2 and $2.5 \times 10^3$ cells $s^{-1}$. Data were analysed as single-parameter DNA frequency histograms using in-house computer software.

**Figure 1** **a**, A photomicrograph of CEM clone C7A cells treated for 54 h with dexamethasone, stained with $1\,\mu g\,ml^{-1}$ acridine orange and viewed by confocal microscopy to obtain a rather sharper image than that from a fluorescent microscope. Apoptotic cells have condensed chromatin (arrow), often arranged in a hemilunar crescentic mass at the nuclear margin (CON = controls; DEX = dexamethasone). **b**, Electrophoresis in agarose of the DNA from clone C7A cells treated for 54 h with dexamethasone showed only a weak classical 'laddering' pattern of DNA cleaved at approximately 200 bp integers. (M = molecular weight markers; C = control cells and at 54 h after dexamethasone treatment). **c**, Pulsed-field electrophoresis of DNA from clone C7A cells treated with dexamethasone for various times. (C = controls; D = 48 h, E = 54 h and F = 60 h after dexamethasone; M = molecular weight markers; Y = yeast artificial chromosome fragments).

### Results

### *Morphological and biochemical features of CCRF CEM clone C7A cell apoptosis*

Figure 1a shows the morphological features of 5 μM dexamethasone-induced apoptosis in clone C7A cells. Typically, fluorescence microscopy showed that apoptotic cells contained discrete masses of condensed chromatin while membrane integrity was maintained at >95% as measured by the exclusion of trypan blue. With time, membrane integrity was lost and the cells then appeared to be swollen and necrotic. Morphological analysis allowed quantitative kinetic estimations of the rate of onset of apoptosis; this type of analysis has been absent from other studies, in which cell lysis and loss of membrane integrity have been estimated. In our hands, the loss of membrane integrity was an event occurring after the appearance of condensed chromatin.

When the numbers of plasma membrane-intact cells with morphological features of chromatin condensation was maximum (>80%), isolation of DNA from these cells showed that it had undergone a modest degree of internucleosomal cleavage typical of apoptosis, presumably after activation of an endonuclease (Figure 1b). Despite intensive efforts only a very weak 'chromatin ladder' typical of the internucleosomal cleavage of DNA to 180–200 bp fragments was observed. However, pulsed-field electrophoresis, which allows the resolution of larger fragments of DNA, showed the early appearance of approximately 300 kbp DNA fragments at times corresponding to the appearance of condensed chromatin (Figure 1c), although this was accompanied by a general fragmentation to lower molecular weights. The loss of the 300 kbp fragment with time and the increase in intensity of the smaller fragments was an event similar to that observed by us in mesenchymal and epithelial cells undergoing apoptosis (Oberhammer *et al.*, 1993) and in thymocytes (Brown *et al.*, 1993). This is discussed below.

### *Kinetics of dexamethasone-induced apoptosis*

Continuous incubation of clone C7A cells with 5 μM dexamethasone induced apoptosis with the kinetics shown in Figure 2a. Previous experiments by us, using a range of concentrations of the steroid, had established that over a 72 h period a 5 μM concentration of dexamethasone induced maximal apoptosis with minimal loss of membrane integrity by 72 h (data not shown). For example, treatment with 1μM dexamethasone induced less than 40% apoptosis by 48 h, whereas this was >55% with 5 μM dexamethasone. During the first 36 h of incubation with 5 μM dexamethasone, at which time there were no apoptotic cells, cell numbers increased as shown in Figure 2b, with little change in the rate of cell division. After 36 h there was no further increase in cell number in the treated cells and only thereafter did cells begin to accumulate in the $G_1$ phase of the cell cycle (see below). Removal of the dexamethasone at any time before 36 h did not result in apoptosis above the control levels over the next 36 h, confirming similar observations by Yuh & Thompson (1989), who used trypan blue to assess viability. Continued incubation with dexamethasone after the initial 36 h of exposure resulted in an accumulating amount of apoptosis over the next 36 h (total exposure 72 h). A 54 h total drug exposure (36 h + 18 h) was selected as an optimal time at which to measure significant changes in apoptosis, since at this time there was minimal loss of membrane integrity with high numbers of apoptotic cells (Figure 2a). We suggest that the first 36 h of exposure to dexamethasone may be equivalent to a 'priming' period, after which the cells became committed to apoptosis on further exposure to the drug. This idea is supported by data from experiments with pulsed incubations with cycloheximide, discussed below.

### *Changes in cell cycle*

It has been shown previously that dexamethasone-induced cell death occurs in the $G_1$ phase of the cell cycle of CCRF CEM cells (Harmon *et al.*, 1979). Analysis of the cell cycle during the course of drug exposure showed that there was no change after 24 h of incubation with dexamethasone, with 56.5 ± 3.6% ($n = 3$, ± s.e.m.) in the $G_1$ phase. The proportion of $G_1$ phase cells had increased only slightly by 36 h to 62.5%, at which time the cells had completed the 'priming' phase but had not yet undergone apoptosis. Only thereafter did the proportion of cells which accumulated in $G_1$ rise: by

**Figure 2** **a**, Kinetics of the onset of cell death in clone C7A cells continuously treated with 5 μM dexamethasone and observed, by microscopy at each time point, in the presence of either 2 μg ml$^{-1}$ acridine orange or 0.2% trypan blue. ▒, trypan blue-positive cells; ■, cells with condensed chromatin ('apoptotic') (means of at least three experiments, ± s.e.m). **b**, Growth kinetics under identical conditions to **a** (s.e.m. <5%, $n = 3$). **c**, Representative Western blot of the cellular content, at various times, of c-*myc* protein during continuous incubation of clone C7A cells with 5 μM dexamethasone: C = control.

21.5% to 78% at 48 h and by 25.6% to 82% by 54 h. Hypodiploid material, representing apoptotic cells, was gated out from each of these analyses.

### *Changes in the expression of* c-myc *protein*

The changes in cellular levels of the c-*myc* protein during incubation with dexamethasone are shown in Figure 2c and are in good agreement with the estimation of changes in the cellular amount of c-*myc* RNA, which fell dramatically after 12 h, as also reported by Yuh & Thompson (1989) under almost identical conditions, but using 1 μM dexamethasone. The rapid disappearance of protein reflects the very short half-life of the c-*myc* protein (*c.* 0.5 h) (Waters *et al.*, 1991).

### *Inhibition of apoptosis and reduction of* c-myc *protein by cycloheximide*

Cycloheximide (CHX) (178 nM) has been shown to partially inhibit glucocorticoid-induced apoptosis in CCRF CEM cells (Bansal *et al.*, 1991). At concentrations $>1$ μM we found it to induce significant ($>50\%$ by 48 h) apoptosis, as has been reported by others (Collins *et al.*, 1991). We found that concentrations less than 1 μM for 48 h did not induce apoptosis. Using pulsed exposures to cycloheximide, we attempted to determine whether there are times during the first 36 h of dexamethasone treatment, and thereafter, during which cells are sensitive to the effects of an inhibitor of protein synthesis. We also wondered what effect cycloheximide would have on changes in the c-*myc* protein content of the cells. There was a profound inhibition of apoptosis when CHX was present during the whole of first 36 h 'priming' period of exposure to dexamethasone (Figure 3). Treatment with cycloheximide during the period *after* c-*myc* protein had become undetectable (from 18 to 54 h) significantly inhibited apoptosis. Most interestingly, incubation with CHX after the initial 36 h exposure to dexamethasone, i.e. at a time when the cells were 'primed' for apoptosis, had no effect on the amount of apoptosis observed at 54 h. It should be noted that during this cycloheximide-insensitive period, there was a continued requirement for dexamethasone (see Figure 2).

CHX treatment for the first 12 h, in the presence or absence of dexamethasone, completely inhibited cell growth, with no increase in cell numbers, and an immediate ($<1$ h) loss of c-*myc* protein was observed. This did not induce apoptosis. First, this result allows us to confirm that this low concentration of cycloheximide effectively inhibits protein synthesis. Secondly, the immediate loss of c-*myc* protein, in the presence or absence of dexamethasone, was not by itself sufficient to induce apoptosis in CEM C7A cells. This will be discussed below. Western blotting for the c-*myc* protein after the removal of cycloheximide showed that the protein reappeared by 2 h and was clearly superinduced under these conditions by 4 h (Figure 4).

### *Kinetics of the loss of a 'memory' of dexamethasone exposure: comparison with re-expression of* c-myc *protein*

To determine whether the events which had occurred during the first 36 h of dexamethasone exposure, which had not actually allowed the cells to engage in apoptosis but had 'primed' the cells (see above), were rapidly reversed, or were retained as a 'memory' of drug exposure, clone C7A cells were exposed to dexamethasone for 36 h (the 'priming' period) and then washed free of drug. A second period of 18 h exposure to dexamethasone (i.e. 54 h exposure in total) was imposed after drug-free periods ranging from zero to 48 h. Normally, 36 h plus 18 h of continuous dexamethasone treatment induced 60% apoptosis (Figure 2). The ability of the cells to undergo apoptosis declined as the period of drug-free exposure after 'priming' was extended (Figure 5a). Thus, the dexamethasone-treated cells lost the 'memory' of their initial 36 h exposure to dexamethasone. We were particularly interested in the kinetics of the reappearance of the c-*myc* protein in comparison with this time-dependent fall in the susceptibility of the cells to apoptosis as memory of the 'priming' events was lost. Figure 5b shows that c-*myc* protein was not detectable by Western blotting until 24 h, by which time $>50\%$ the total cell population had already become resistant to the induction of apoptosis on readdition of dexamethasone. Most pertinently, after 12 h of a drug-free incubation and subsequent readdition of dexamethasone, apoptosis had fallen from 60% to 20%, although no c-*myc* protein was detectable at 12 h. Interestingly, incubation with CHX during the drug-free period maintained some of the sensitivity of the cells to subsequent readdition of dexamethasone for 18 h (Figure 5) and so inhibited the loss of memory of the events initiated during 'priming'. This will be discussed below.

**Figure 3** The effects of various periods of incubation with 200 nM cycloheximide (CHX) (solid bars, bottom) on the percentages of apoptotic cells at 54 h during continuous treatment with 5 μM dexamethasone. Top: The kinetic profile of the onset of apoptosis. The vertical line arbitrarily divides the profile of apoptosis into a 0–36 h 'priming' phase and an 18 h 'commitment' phase (means of three experiments with s.e.m. $<5\%$).

### Discussion

The kinetics of cell death of human CCRF CEM clone C7A lymphoid cells, induced by dexamethasone, has been defined in detail here. Although, using morphological criteria, the cells appeared to be apoptotic with high percentages of cells having an intact plasma membrane and features typical of chromatin condensation (Figure 1a), we were unable to obtain good evidence of one of the cardinal biochemocal features of apoptosis, the DNA 'ladder' (Figure 1b), indicative of internucleosomal cleavage by an endonuclease. Analysis of DNA integrity by pulsed-field electrophoresis showed that after 48 h of dexamethasone approximately

**Figure 4** Western blot of the restoration, with time, of c-*myc* protein content of CCRF CEM clone C7A cells after incubation with 200 nM cycloheximide for 36 h (no DEX present in this experiment).

**Figure 5** **a**, Kinetic analysis of the rate of decline of apoptosis in clone C7A cells which had been treated with 5 μM dexamethasone for 36 h, the dexamethasone removed by washing, and the cells incubated for increasing periods of time (*x*-axis) in fresh medium (without dexamethasone) prior to the readdition of 5 μM dexamethasone for 18 h. This gave a total exposure time to dexamethasone of 54 h, at which time the numbers of apoptotic cells were estimated by microscopy, as detailed in Materials and methods. CHX = 200 nM CHX was added during the intervening dexamethasone-free periods. **b**, Western blot analysis of the cellular amount of c-*myc* protein after a 36 h exposure of cells to 5 μM dexamethasone, at which point the cells were washed free of the drug and harvested at the times shown.

300 kbp fragments of DNA were present (Figure 1c). This disappeared with time and diffuse bands of below 50 kbp appeared. Work by Walker *et al.* (1991) has shown that dexamethasone induces the appearance of the 300 kbp fragments in thymocytes, and it was suggested that they may represent domains of chromatin loops becoming detached from their anchoring points on the nuclear matrix. These looped domains in apoptotic thymocytes then resolved into approximately 50 kbp fragments. Clone C7A CEM cells consistently formed fragments of lower molecular weight than this and there appeared to be much non-specific DNA cleavage. Recent work by us (Oberhammer *et al.*, 1993) has shown that these large DNA fragments, which are not resolved by standard agarose gel electrophoresis, are observed prior to and sometimes in the absence of internucleosomal fragmentation to 180–200 bp integer DNA fragments, typical of the DNA 'ladder'. We suggest that apoptotic cells be identified by morphological features of chromatin condensation and that the appearance of high molecular weight DNA fragments is a more reliable biochemical characteristic of condensed chromatin, in contrast to the appearance of internucleosomal fragments. It is interesting that such findings were made in a cell type of haematopoietic origin, in which the typical endonucleolytic cleavage of DNA has been considered to be the hallmark of apoptosis.

In contrast to immature mouse thymocytes treated with dexamethasone, in which the onset of apoptosis is rapid (*c.* 6 h) (Wyllie, 1980) the onset of apoptosis in clone C7A cells did not occur until after 36 h of drug exposure (Figure 2). During this 36 h, changes were presumably occurring that were essential for the subsequent expression of apoptosis since removal of dexamethasone at any time before this failed to induce apoptosis thereafter. This was commented on previously by Yuh & Thompson (1989). The extended period of drug exposure, prior to the onset of changes in chromatin that heralds the death of the cell, is we suggest similar to the precommitment periods observed in cells induced to terminally differentiate after drug treatment (Yen *et al.*, 1984; Yen 1985). We equate this first 36 h with a period of 'priming' (Dive & Wyllie, 1993) of the cells for the subsequent second phase of the engagement of apoptosis. The separation of these two phases potentially allows for a discrete dissection of the suppression or activation of gene expression, and protein synthesis, required for apoptosis.

Evidence from transient transfection experiments, which utilised deletion constructs of the glucocorticoid receptor, suggests that transcriptional suppression is necessary for the death of clone C7A cells (Nazareth *et al.*, 1991). Indeed, Yuh & Thompson (1989) showed an early loss of c-*myc* RNA in dexamethasone-treated clone C7A cells, confirmed here at the protein level (Figure 2c). The loss of the c-*myc* protein may contribute to the loss of some essential *myc*-associated transcripts necessary for cell survival. The fall in c-*myc* protein occurred at a time (12 h) prior to any change in the cell cycle of the cells (> 36 h) (see Results) and, while it is generally considered that the expression of c-*myc* reflects the proliferative status of the cell population (Einat *et al.*, 1985, Waters *et al.*, 1991), its absence did not inhibit the continued proliferation of dexamethasone-treated clone C7A cells for a period which corresponded to another full cell doubling (Figure 2b). It has recently been reported that differentiating agents reduced synthesis of c-*myc* RNA to undetectable levels in HL-60 cells, yet these cells also underwent a full further round of cell division (Mitchell *et al.*, 1992; Beere *et al.*, 1993*a*). Thus, a key 'priming' event is not a change in cell proliferation *per se*, driven by a fall in c-*myc* expression. Indeed, conditions of the complete inhibition of cell growth by cycloheximide, accompanied by a fall in c-*myc* protein (Figure 4), inhibited cell death induced by dexamethasone (Figure 3).

With respect to the idea that transcriptional repression, including that of c-*myc*, is essential for the onset of apoptosis, Figure 3 shows that cycloheximide (CHX) completely inhibited apoptosis if it was present during the first 36 h of dexamethasone treatment. This is strongly suggestive of a role for new protein synthesis during the 'priming' period, presumably after transcriptional activation by the glucocorticoid, since functionally active glucocorticoid receptors are required for the death of these cells (Harmon & Thompson, 1981). These observations suggest that activational events, including protein synthesis, were taking place, which subsequently allowed the 'machinery' of cell death to be engaged thereafter. Presumably the synthesis of new proteins, inhibited by CHX, had followed transcriptional activation by dexamethasone via a glucocorticoid-responsive element. Incubation of CCRF CEM clone C7A cells with 200 nM CHX for 36 h also rapidly (< 1 h) inhibited the expression of c-*myc* protein, an event suggested to be a critical harbinger of dexamethasone-induced apoptosis (Thulasi *et al.*, 1993), but did not induce apoptosis, suggesting that events in addition to the loss of c-*myc* are critical to the engagement of apoptosis in these cells, an idea that was supported by the observation that CHX inhibited apoptosis even after the suppression of c-*myc* protein had occurred (> 18 h) (Figure 3).

Once the cells had become 'primed', during the first 36 h of dexamethasone tretment, apoptosis then proceeded independently of new protein synthesis, since it was CHX insensitive (Figure 3). As the continued presence of dexamethasone was required for apoptosis during the period which followed 'priming' (i.e. after 36 h) (Figure 2) transcriptional suppression, mediated via a glucocorticoid-responsive element, was presumably the dominant event occurring in this second phase.

Cycloheximide completely inhibited cell growth (Results), and might have prevented apoptosis because of this or, more subtly, by preventing progression through some discrete 'window' of the cell cycle essential for the engagement of apoptosis. It should be noted that after 'priming' (i.e. after 36 h of dexamethasone) cells were moving out of cycle, with an accumulating number in $G_1$ phase (see Results), but were not out of cycle. Yet cycloheximide had no effect on apoptosis during the 'post-priming' period. It is possible that by 36 h all of the cells had passed through this 'window' of the cell cycle, necessary for them to later engage in apoptosis, and that cycloheximide inhibited cell death in the first 36 h by preventing passage through such a 'window'. Such a hypothesis awaits provision of populations of cell cycle-synchronised cells.

Changes which occurred in the 'priming' period, requiring protein synthesis after transcriptional activation, remained as a 'memory' during the following 36 h (Figure 5). The partial maintenance of this memory by CHX (Figure 5) suggests that messenger RNAs are possibly stabilised during this time. Analysis of the kinetics of changes in gene expression, and other events, during the 'priming' phase together with, and compared with, the kinetics of the decay of memory, with or without CHX, will be helpful in delineating the temporal hierarchy of events leading to the commitment to death of a cell. We plan to tackle this by a subtractive hybridisation approach (Brady *et al.*, 1991). It has already been commented upon that the loss of c-*myc* protein from CEM clone C7A cells was not an event associated with full commitment to apoptosis, since dexamethasone treatment had to be continued for at least another 24 h before the cells became committed to die and removal of dexamethasone prior to this abrogated subsequent cell death (Figure 2 and Results). This was mirrored in the kinetics of memory decay: c-*myc* protein was not detectable until 24 h after dexamethasone removal. Thus, from the combined data of Figures 2, 3 and 5 we suggest that it is probable that an accumulation of events before and after the suppression of c-*myc* expression is required before cells can become committed to die. That events before the loss of c-*myc* expression may be essential was suggested by the observation that as the memory of exposure to dexamethasone decayed (Figure 5) cells became refractive to dexamethasone-induced apoptosis *before* re-expression of detectable levels of c-*myc* protein. Thulasi *et al.* (1993) recently reported that transient transfection of these cells with either inducible or constitutively expressed c-*myc*-containing constructs inhibited dexamethasone-induced cell death. While this supports the idea that c-*myc* suppression is involved in the apoptosis of these cells, our data suggest that it is certainly not sufficient for commitment of clone C7A cells to an apoptotic death.

Recent work in Rat-1 fibroblasts has shown that artefactually deregulated expression of c-*myc* also predisposes the cells to die by apoptosis when they are grown under conditions limiting to proliferation and survival (Evan *et al.*, 1992). Analogous findings in myeloid and T cells have been reported (Askew *et al.*, 1991; Shi *et al.*, 1992). Even *et al.* (1992) have suggested that, in addition to the role of c-*myc* in regulating cell proliferation, its capability to induce apoptosis may act as a safeguard if cell proliferation occurs independently of the appropriate growth factor signals. This idea presumes that truly deregulated expression does take place under these conditions. In support of this, earlier studies by Wyllie *et al.* (1987) had shown that apoptotic rates in transplanted fibroblasts were greater after transfection with the c-*myc* gene and the ease of induction of apoptosis in a number of different cell lines after drug treatment appeared to correlate with their expression of the c-*myc* gene (Bertrand *et al.*, 1991). However, CCRF CEM clone C7A cells do not appear to conform to this model since c-*myc* protein levels fell prior to the onset of apoptosis with no evidence of a rise before the onset of apoptosis. We have also recently shown that HL-60 cells, which have an amplified c-*myc* gene, down-regulate both c-*myc* RNA and protein prior to apoptosis (Beere *et al.*, 1993*b*).

The kinetics of the induction of cell death, the formation of a 'memory' of exposure to dexamethasone and the kinetics of decay of this memory, modified by CHX treatment, as described here, promote the CCRF CEM clone C7A model as being valuable for further studies of the genetic changes that are required to bring about drug-induced cell death. Careful comparisons of the kinetics of dexamethasone-induced cell death, and the associated changes in gene expression, with those of cell death induced by agents with different targets within the cell should allow the determination of the generality of the events which are necessary for the induction of cell death. Knowing these may promote strategies for the induction of cell death in chemoresistant tumours (Hickman, 1992).

Alan Wood was supported by a joint studentship from the Science and Engineering Research Council and Zeneca Pharmaceuticals; Catherine Waters was supported by the Wellcome Trust and John Hickman was supported by Program Grant SP 2115 from the Cancer Research Campaign. We thank Gerard Evan for antibody and Caroline Dive for criticism of the manuscript.

### References

ASKEW, D.S., ASHMUN, R.A., SIMMONS, B.C. & CLEVELAND, J.L. (1991). Constitutive c-myc expression in an IL-3-dependent myeloid cell line suppresses cell cycle arrest and accelerates apoptosis. *Oncogene*, **6**, 1915–1922.

BANSAL, N., HOULE, A.G. & MELNYKOVYCH, G. (1991). Apoptosis: mode of cell death induced in T cell leukemia lines by dexamethasone and other agents. *FASEB J.*, **5**, 211–216.

BEERE, H.M., MORIMOTO, R.I. & HICKMAN, J.A. 1993*a*). Investigations of mechanisms of drug-induced changes in gene expression: *N*-methylformamide-induced changes in the synthesis of the $M_r$ 72,000 constitutive heat shock protein during commitment of HL-60 cells to granulocytic differentiation. *Cancer Res.*, **53**, 3034–3039.

BEERE, H.M., HICKMAN, J.A., MORIMOTO, R.I., PARMAR, R., NEWBOULD, R. & WATERS, C.M. (1993*b*). Changes in hsc 70 and c-*myc* in HL-60 cells engaging differentiation or apoptosis. *Mol. Cell Different.*, **1**, 323–343.

BERTRAND, R., SARANG, M., JENKIN, J., KERRIGAN, D. & POMMIER, Y. (1991). Differential induction of secondary DNA fragmentation by topoisomerase II inhibitors in human tumor cell lines with amplified c-*myc* expression. *Cancer Res.*, **51**, 6280–6285.

BRADY, G., BARBARA, M. & ISCOVE, N.N. (1991). Amplified representative cDNA libraries from single cells. *Meth. Mol. Cell Biol.*, **2**, 17–25.

BROWN, D.G., SUN, X.-M. & COHEN, G.M. (1993). Dexamethasone-induced apoptosis involves cleavage of DNA to larger fragments prior to internucleosomal fragmentation. *J. Biol. Chem.*, **268**, 3037–3039.

COLE, M.D. (1991). Myc meets its Max. *Cell*, **65**, 715–716.

COLLINS, R.J., HARMON, B.V., SOULVIS, T., POPE, J.H. & KERR, J.F.R. (1991). Effects of cycloheximide on B-chronic lymphocytic leukemia and normal lymphocytes *in vitro*: induction of apoptosis. *Br. J. Cancer*, **64**, 518–522.

DIVE, C. & WYLLIE, A.H. (1993). Apoptosis and cancer chemotherapy. In: *Frontiers in Pharmacology: Cancer Chemotherapy*, Hickman, J.A. & Tritton, T.R. (eds), pp. 21–55. Blackwell Scientific Publications: Oxford.

DIVE, C., GREGORY, C.D., PHIPPS, D.J., EVANS, D.L., MILNER, A.E. & WYLLIE, A.H. (1992). Analysis and discrimination of necrosis and apoptosis (programmed cell death) by multiparameter flow cytometry. *Biochim. Biophys. Acta*, **1133**, 275–285.

EILERS, M., SCHIRM, S. & BISHOP, J.M. (1991). The MYC protein activates transcription of the α-prothymosin gene. *EMBO J.*, **10**, 133–141.

EINAT, E., RESNITSKY, D. & KIMCHI, A. (1985). Close link between reduction of c-*myc* expression by interferon and $G_0/G_1$ arrest. *Nature*, **313**, 597–600.

EVAN, G.I., LEWIS, G.K., RAMSEY, G. & BISHOP, J.M. (1985). Isolation of monoclonal antibodies specific for human c-myc protooncogene product. *Mol. Cell. Biol.*, **5**, 3610-3616.

EVAN, G.I., WYLLIE, A.H., GILBERT, C.S., LITTLEWOOD, T.D., LAND, H., BROOKS, M., WATERS, C.M., PENN, L.Z. & HANCOCK, D.C. (1992). Induction of apoptosis in fibroblasts by c-myc protein. *Cell*, **69**, 119-128.

HARMON, J.M. & THOMPSON, E.B. (1981). Isolation and characterization of dexamethasone-resistant mutants from human lymphoid cell line CEM-7. *Mol. Cell Biol.*, **1**, 512-521.

HARMON, J.M., NORMAN, M.R., FOWLKES, B.J. & THOMPSON, E.B. (1979). Dexamethasone induces irreversible $G_1$ arrest and death of a human lymphoid cell line. *J. Cell. Physiol.*, **98**, 267-278.

HICKMAN, J.A. (1992). Apoptosis induced by anticancer drugs. *Cancer Metast. Rev.*, **11**, 121-139.

MITCHELL, L.S., NEIL, R.A. & BIRNIE, G.D. (1992). Temporal relationships between induced changes in c-myc mRNA abundance, proliferation and differentiation in HL60 cells. *Differentiation*, **49**, 119-125.

NAZARETH, L.V., HARBOUR, D.V. & THOMPSON, E.B. (1991). Mapping the human glucocorticoid receptor for leukemic cell death. *J. Biol. Chem.*, **266**, 12976-12980.

NORMAN, M.R. & THOMPSON, E.B. (1977). Characterization of a glucocorticoid sensitive human lymphoid cell line. *Cancer Res.*, **37**, 3875-3791.

OBERHAMMER, F., WILSON, J., DIVE, C., MORRIS, I.D., HICKMAN, J.A., WAKELING, A.E., WALKER, P.R. & SIKORSKA, M. (1993). Apoptotic death in epithelial cells: cleavage of DNA to 300 and/or 50 kb fragments prior to or in the absence of internucleosomal fragmentation. *EMBO J.*, **12**, 3679-3684.

OWENS, G.P. & COHEN, J.J. (1992). Identification of genes involved in programmed cell death. *Cancer Metast. Rev.*, **11**, 149-156.

SHI, Y., GLYNN, J.M., GULBERT, L.J., COTTER, T.G., BISSONETTE, R.P. & GREEN, D.R. (1992). Role for c-myc in activation induced apoptotic cell deaths in T cell hybridomas. *Science*, **257**, 212-214.

THULASI, R., HARBOUR, D.V. & THOMPSON, E.B. (1993). Suppression of c-*myc* is a critical step in glucocorticoid-induced human leukemic cell lysis. *J. Biol. Chem.*, **268**, 18306-18312.

TOWBIN, H., STAEHLIN, T. & GORDON, J. (1989). Electrophoretic transfer of proteins from polyacrylamide gels to nitrocellulose sheets; procedures and some applications. *Proc. Natl Acad. Sci. USA*, **76**, 4350-4354.

WATERS, C.M., LITTLEWOOD, T.D., HANCOCK, D.C., MOORE, J.P. & EVAN, G.I. (1991). C-myc protein expression in untransformed fibroblasts. *Oncogene*, **6**, 797-805.

WALKER, P.R., SMITH, C., YOUDALE, T., LEBLANC, J., WHITFIELD, J.F. & SIKORSKA, M. (1991). Topoisomerase II-reactive chemotherapeutic drugs induce apoptosis in thymocytes. *Cancer Res.*, **51**, 1078-1085.

WYLLIE, A.H. (1980). Glucocorticoid-induced thymocyte apoptosis is associated with endogenous endonuclease activation. *Nature*, **284**, 555-556.

WYLLIE, A.H., ROSE, K.A., MORRIS, R.G., STEEL, C.M., FOSTER, E. & SPANDIDOS, D.A. (1987). Rodent fibroblast tumours expressing human myc and ras genes: growth metastasis and endogenous oncogene expression. *Br. J. Cancer*, **56**, 251-259.

YEN, A. (1985). Control of HL-60 myeloid differentiation. *Exp. Cell Res.*, **156**, 198-212.

YEN, A., REECE, S.A. & ALBRIGHT, K.L. (1984). Dependence of HL60 myeloid cell differentiation on continuous and split retinoic acid exposures; precommitment memory associated with altered nuclear structure. *J. Cell Physiol.*, **118**, 277-286.

YUH, Y.-S. & THOMPSON, E.B. (1989). Glucocorticoid effect on oncogene/growth gene expression in human T lymphoblastic leukemic cell line CCRF-CEM. *J. Biol. Chem.*, **264**, 10904-10910.